# Incremental Impact of Standard Modifiable Cardiovascular Risk Factors on Long-Term Outcomes After Acute Myocardial Infarction

**DOI:** 10.1016/j.jacasi.2026.03.030

**Published:** 2026-05-06

**Authors:** Shogo Okita, Yuichi Saito, Hiroaki Yaginuma, Osamu Hashimoto, Takanori Sato, Hideki Kitahara, Yoshio Kobayashi

**Affiliations:** a Department of Cardiovascular Medicine, Chiba University Hospital, Chiba, Japan; b Department of Cardiology, Chiba Emergency and Psychiatric Medical Center, Chiba, Japan

**Keywords:** acute myocardial infarction, diabetes, dyslipidemia, hypertension, smoking

## Abstract

**Background:**

The lack of standard modifiable cardiovascular risk factors (SMuRFs), including hypertension, diabetes, dyslipidemia, and smoking, is linked to worse in-hospital outcomes in acute myocardial infarction (AMI). Whether the number of SMuRFs is associated with long-term outcomes after AMI remains unclear.

**Objectives:**

The authors aimed to evaluate the incremental prognostic impact of the number of SMuRFs on outcomes after discharge in patients with AMI.

**Methods:**

This multicenter, retrospective registry included 2,059 patients with AMI who underwent primary percutaneous coronary intervention. Patients were categorized according to the number of SMuRFs (0-4). The primary endpoint was major adverse cardiovascular events (MACEs), a composite of cardiovascular death, heart failure rehospitalization, recurrent AMI, and ischemic stroke. In addition, we assessed the relative prognostic impact among the 4 SMuRFs.

**Results:**

Of the 2,059 patients, 96 (4.7%), 489 (23.7%), 779 (37.8%), 562 (27.3%), and 133 (6.5%) had 0, 1, 2, 3, and 4 SMuRFs, respectively. During the median follow-up period of 538 (IQR: 349-1316) days, 210 of 2,059 (10.2%) patients experienced an MACE. The overall risk of MACE did not differ across the groups, whereas the number of SMuRFs was progressively associated with an increased risk of recurrent AMI. Among the 4 SMuRFs, hypertension was independently associated with MACE in multivariable analysis (adjusted HR: 1.629; 95% CI: 1.117-2.376).

**Conclusions:**

The long-term cardiovascular risk after discharge did not differ significantly by the number of SMuRFs in patients with AMI. Among the 4 SMuRFs, hypertension was associated with an increased risk of an MACE.

Atherosclerotic cardiovascular diseases, represented by acute myocardial infarction (AMI), are mainly attributable to standard modifiable cardiovascular risk factors (SMuRFs), including hypertension, diabetes, dyslipidemia, and current smoking.^1^ Targeted management of such risk factors is recommended in the current guidelines and can improve cardiovascular outcomes in the primary and secondary prevention settings.^2^, ^3^, ^4^, ^5^ Nonetheless, a substantial proportion of patients present with AMI in the absence of SMuRFs, with the reported prevalence ranging from 2% to 31%.^6^ In previous observational studies and meta-analyses, SMuRF-less patients with AMI reportedly and counterintuitively had worse short-term clinical outcomes.^7^, ^8^, ^9^, ^10^, ^11^, ^12^ In addition, some previous studies have reported an excess long-term cardiovascular risk in SMuRF-less patients,^13^, ^14^, ^15^ whereas in other studies, the lack of SMuRFs had a neutral or positive effect on the prognosis after AMI.^8^^,^^16^^,^^17^ Given the accumulated risks for atherosclerotic cardiovascular events,^5^ the incremental burden of SMuRFs may contribute to poor long-term outcomes after AMI. Indeed, in patients with chronic coronary artery disease in the CLARIFY (ProspeCtive observational LongitudinAl RegIstry oF patients with stable coronary arterY disease) registry, the cumulative incidence of death or AMI at 5 years was progressively increased according to the number of risk factors, with 5.4%, 6.0%, 7.3%, 9.0%, and 9.7% in patients having 0, 1, 2, 3, and 4 SMuRFs, respectively.^18^ The accumulation of traditional risks presumably leads to a worse prognosis, and intensive secondary prevention based on the burden of SMuRFs may improve clinical outcomes. The prognostic impact of the absence of SMuRFs has been recently investigated, whereas whether the dose-response relationship is present in patients with AMI remains uncertain. In the present study, we evaluated the association between the number of SMuRFs and long-term cardiovascular outcomes after AMI.

## Methods

### Study design

**Figure 1:**
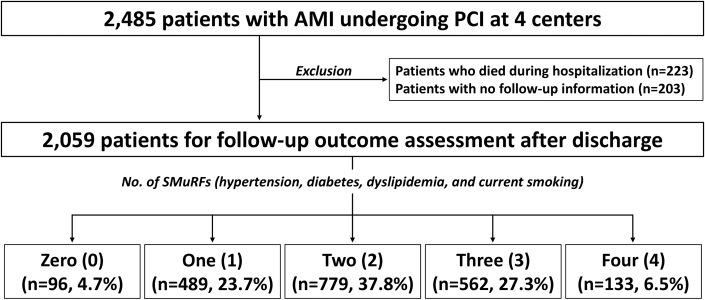
Study Flow A total of 2,059 patients with AMI undergoing PCI were included in the present study and were divided into 5 groups according to the number of SMuRFs, such as hypertension, diabetes, dyslipidemia, and current smoking. AMI = acute myocardial infarction; PCI = percutaneous coronary intervention; SMuRF = standard modifiable cardiovascular risk factor.

This was a retrospective, multicenter registry study.^19^^,^^20^ From January 2012 to December 2021, a total of 2,485 patients with AMI underwent primary percutaneous coronary intervention (PCI) at 4 tertiary referral hospitals in Japan, according to local standard practice, including the use of dual antiplatelet therapy, intracoronary imaging, contemporary drug-eluting stents, and mechanical circulatory support if needed.^21^, ^22^, ^23^, ^24^, ^25^, ^26^, ^27^ Overall, the clinical management was left to the discretion of the treating physicians in a real-world setting. AMI included ST-segment elevation myocardial infarction (STEMI) and NSTEMI and was defined based on the fourth universal definition.^28^ ST-segment deviation, which is a component of the guideline-endorsed GRACE (Global Registry of Acute Coronary Events) risk score and is associated with poor outcomes in patients with NSTEMI, was also evaluated.^29^^,^^30^ After excluding patients who died during hospitalization (n = 223) and those with no follow-up information (n = 203), 2,059 were included in the present study (Figure 1). This study was done in accordance with the Declaration of Helsinki and was approved by the ethics committee of each hospital. Informed consent for this study was obtained in an opt-out manner.

### Definitions

In the present study, SMuRFs included hypertension, diabetes, dyslipidemia, and current smoking.^11^^,^^12^ Hypertension was defined as having a previous diagnosis of hypertension or previous antihypertensive pharmacological treatment, or a new diagnosis of hypertension during hospitalization with systolic blood pressure ≥140 mm Hg and/or diastolic blood pressure ≥90 mm Hg. Diabetes was defined as a previous diagnosis, previous glucose-lowering medications, or hemoglobin A1c ≥6.5%. Dyslipidemia was defined as a previous diagnosis, previous pharmacological treatment, low-density lipoprotein cholesterol ≥140 mg/dL, high-density lipoprotein cholesterol <40 mg/dL, or fasting triglycerides >150 mg/dL. Current smoking was defined as a history of tobacco smoking within the past year.^21^ Patients were divided into 5 groups according to the number of SMuRFs, ranging from 0 to 4 (Figure 1).

### Outcomes

The follow-up data were obtained from the medical records of each institution. The primary endpoint was major adverse cardiovascular events (MACEs), a composite of cardiovascular death, heart failure rehospitalization, recurrent AMI, and ischemic stroke, adjudicated according to the consensus document.^31^ We primarily evaluated the relationship between the number of SMuRFs and clinical outcomes after discharge in patients with AMI. Each component of MACE was also assessed. In addition, we explored the prognostic impact of individual SMuRFs and their combinations.

### Statistical analysis

Statistical analyses were performed using R software version 4.3.1. (The R Foundation for Statistical Computing). Data are presented as mean ± SD, median [IQR], or frequency with percentage, as appropriate. Continuous variables were analyzed using analysis of variance and the Kruskal-Wallis test. Categorical variables were evaluated with Fisher's exact test. Kaplan-Meier analysis with log-rank test was performed to calculate the time to the primary outcome events after discharge, with landmark analysis using the date of discharge as a landmark. For the outcome events, the incidence rates were evaluated as being crude and based on 3-year Kaplan-Meier estimates with 95% CIs. Univariable and multivariable analyses were performed using Cox proportional hazards models to estimate unadjusted and adjusted HRs with corresponding 95% CIs. Age, sex, body mass index, previous heart failure, previous myocardial infarction, left ventricular ejection fraction, estimated glomerular filtration rate, hemoglobin, STEMI (vs NSTEMI), cardiogenic shock, and 3-vessel disease, along with the SMuRFs, were included in the multivariable models.^7^^,^^29^^,^^32^ The use of mechanical circulatory support devices, cardiac arrest, and levels of glycated hemoglobin and low-density lipoprotein cholesterol were not included in the multivariable model due to the confounding effect with other variables. The effect of combinations of SMuRFs was also evaluated in the multivariable analysis. The proportional hazards assumption was evaluated using Schoenfeld residuals and was satisfied for the primary exposures, whereas sex, body mass index, and hemoglobin level violated the assumption. Thus, a stratified multivariable Cox model was fitted as a sensitivity analysis, with sex and with body mass index and hemoglobin level stratified at their median values (24.2 kg/m^2^ for body mass index and 14.3 g/dL for hemoglobin level). In addition, sensitivity analyses were performed by sex. A value of *P* < 0.05 was considered statistically significant.

## Results

Of the 2,059 patients with AMI who underwent PCI, 96 (4.7%) had none of the SMuRFs, whereas 489 (23.7%), 779 (37.8%), 562 (27.3%), and 133 (6.5%) patients had 1, 2, 3, and 4 SMuRFs, respectively (Figure 1). The distribution of SMuRF combinations is listed in Supplemental Table 1. Baseline characteristics of each group are shown in Table 1. SMuRF-less patients were older and had lower body mass index than those with ≥1 SMuRFs (Table 1). The prevalence of previous myocardial infarction was lowest, whereas active cancer was most frequent in patients with no SMuRFs (Table 1). Although the patterns of clinical presentation were similar, those of prescription at discharge differed significantly among the 5 groups (Table 1). Procedural characteristics are listed in Table 2. The proportion of multivessel PCIs increased progressively with the number of SMuRFs, from 0 to 4 (Table 2).

**Table 1:** Baseline Characteristics According to the Number of SMuRFs

	All (N = 2,059)	Number of SMuRFs					*P* Value
		0 (n = 96)	1 (n = 489)	2 (n = 779)	3 (n = 562)	4 (n = 133)	
Age, y	67.3 ± 12.4	69.5 ± 14.6	69.2 ± 12.0	68.1 ± 12.4	65.7 ± 12.0	61.5 ± 11.1	<0.001
Men	1,597 (77.6)	72 (75.0)	365 (74.6)	599 (76.9)	437 (77.8)	124 (93.2)	<0.001
BMI, kg/m^2^	24.4 ± 3.9	23.0 ± 3.3	23.6 ± 3.6	24.3 ± 3.5	25.1 ± 4.1	26.9 ± 4.6	<0.001
Hypertension	1,421 (69.0)	0	208 (42.5)	574 (73.7)	506 (90.0)	133 (100)	<0.001
Diabetes	736 (35.7)	0	36 (7.4)	184 (23.6)	383 (68.1)	133 (100)	<0.001
Dyslipidemia	1,357 (65.9)	0	173 (35.4)	539 (69.2)	512 (91.1)	133 (100)	<0.001
Current smoker	751 (36.5)	0	72 (14.7)	261 (33.5)	285 (50.7)	133 (100)	<0.001
Active cancer	86 (4.2)	10 (10.4)	28 (5.7)	24 (3.1)	18 (3.2)	6 (4.5)	0.003
Previous HF	40 (1.9)	1 (1.0)	8 (1.6)	15 (1.9)	14 (2.5)	2 (1.5)	0.796
Previous MI	155 (7.5)	2 (2.1)	20 (4.1)	59 (7.6)	63 (11.2)	11 (8.3)	<0.001
Atrial fibrillation	112 (5.4)	8 (8.3)	38 (7.8)	38 (4.9)	25 (4.4)	3 (2.3)	0.028
MCS device							
IABP	135 (6.6)	6 (6.2)	30 (6.1)	48 (6.2)	44 (7.8)	7 (5.3)	0.698
VA-ECMO	35 (1.7)	3 (3.1)	9 (1.8)	12 (1.5)	10 (1.8)	1 (0.8)	0.721
Echocardiographic data							
LVEF, %	49.1 ± 11.9	49.3 ± 12.9	50.2 ± 12.7	48.2 ± 11.9	49.0 ± 11.1	49.9 ± 11.9	0.058
Electrocardiographic data							
STEMI	1,422 (69.1)	71 (74.0)	334 (68.3)	543 (69.7)	382 (68.0)	92 (69.2)	0.796
NSTEMI	637 (30.9)	25 (26.0)	155 (31.7)	236 (30.3)	180 (32.0)	41 (30.8)	0.796
ST-segment deviation	206 (32.3)	8 (32.0)	55 (35.5)	75 (31.8)	55 (30.6)	13 (31.7)	0.909
eGFR, mL/min/1.73 m^2^	66.5 ± 23.5	66.5 ± 16.8	65.2 ± 21.9	66.5 ± 22.5	66.1 ± 26.0	72.1 ± 27.4	0.053
Hemoglobin, g/dL	14.0 ± 2.1	13.1 ± 2.3	13.7 ± 2.1	14.1 ± 2.0	14.0 ± 2.3	15.1 ± 1.9	<0.001
HbA1c, %	6.4 ± 1.4	5.7 ± 0.4	5.8 ± 0.7	6.2 ± 1.1	7.0 ± 1.7	7.8 ± 1.7	<0.001
LDL-C, mg/dL	123.4 ± 41.1	110.9 ± 33.7	123.3 ± 40.5	126.0 ± 39.2	122.0 ± 44.6	123.4 ± 41.2	0.015
Clinical presentation							
Cardiogenic shock	190 (9.2)	7 (7.3)	47 (9.6)	72 (9.2)	55 (9.8)	9 (6.8)	0.793
Cardiac arrest	125 (6.1)	7 (7.3)	27 (5.5)	52 (6.7)	34 (6.0)	5 (3.8)	0.689
Medications at discharge							
Aspirin	1,903 (92.4)	87 (90.6)	444 (90.8)	716 (91.9)	527 (93.8)	129 (97.0)	0.091
P2Y12 inhibitor	1,978 (96.2)	87 (90.6)	469 (95.9)	747 (95.9)	546 (97.3)	129 (97.0)	0.025
Oral anticoagulation	267 (13.0)	11 (11.5)	86 (17.6)	100 (12.8)	65 (11.6)	5 (3.8)	<0.001
Statin	1,913 (92.9)	81 (84.4)	436 (89.2)	739 (94.9)	529 (94.1)	128 (96.2)	<0.001
SGLT-2 inhibitor	196 (9.5)	1 (1.0)	15 (3.1)	41 (5.3)	97 (17.3)	42 (31.6)	<0.001
Insulin	80 (3.9)	0 (0)	6 (1.2)	24 (3.1)	42 (7.5)	8 (6.0)	<0.001
Calcium-channel blocker	407 (19.8)	9 (9.4)	74 (15.1)	147 (18.9)	144 (25.6)	33 (24.8)	<0.001
ACEI, ARB, or ARNI	1,617 (78.5)	67 (69.8)	356 (72.8)	609 (78.2)	473 (84.2)	112 (84.2)	<0.001
β-blocker	1,537 (74.6)	75 (78.1)	350 (71.6)	578 (74.2)	426 (75.8)	108 (81.2)	0.162
MRA	354 (17.2)	15 (15.6)	82 (16.8)	132 (16.9)	107 (19.0)	18 (13.5)	0.582
Diuretic	378 (18.4)	18 (18.8)	88 (18.0)	139 (17.8)	116 (20.6)	17 (12.8)	0.297

Values are mean ± SD or n (%).

ACEI = angiotensin-converting enzyme inhibitor; ARB = angiotensin II receptor blocker; ARNI = angiotensin receptor neprilysin inhibitor; BMI = body mass index; eGFR = estimated glomerular filtration rate; HbA1c = glycated hemoglobin; HF = heart failure; IABP = intra-aortic balloon pump; LVEF = left ventricular ejection fraction; LDL-C = low-density lipoprotein cholesterol; MCS = mechanical circulatory support; MI = myocardial infarction; MRA = mineralocorticoid receptor antagonist; NSTEMI = non–ST-segment elevation myocardial infarction; SGLT-2 = sodium-glucose cotransporter 2; SMuRF = standard modifiable cardiovascular risk factor; STEMI = ST-segment elevation myocardial infarction; VA-ECMO = veno-arterial extracorporeal membrane oxygenation.

**Table 2:** Procedural Characteristics According to the Number of SMuRFs

	All (N = 2,059)	Number of SMuRFs					*P* Value
		0 (n = 96)	1 (n = 489)	2 (n = 779)	3 (n = 562)	4 (n = 133)	
Culprit vessel							0.196
RCA	677 (32.9)	33 (34.4)	142 (29.0)	256 (32.9)	200 (35.7)	46 (34.6)	
LAD/LMT	1,014 (49.3)	49 (51.0)	265 (54.2)	384 (49.3)	253 (45.1)	63 (47.4)	
LCX	313 (15.2)	13 (13.5)	72 (14.7)	123 (15.8)	85 (15.2)	20 (15.0)	
3-vessel disease	377 (18.3)	13 (13.5)	61 (12.5)	136 (17.5)	141 (25.1)	26 (19.5)	<0.001
Access site							0.668
Radial artery	1,896 (92.1)	91 (94.8)	450 (92.0)	719 (92.3)	510 (90.7)	126 (94.7)	
Femoral artery	136 (6.6)	5 (5.2)	33 (6.7)	48 (6.2)	43 (7.7)	7 (5.3)	
Others	27 (1.3)	0 (0)	6 (1.2)	12 (1.5)	9 (1.6)	0 (0)	
Intracoronary imaging							0.452
IVUS	2,008 (97.5)	91 (94.8)	476 (97.3)	760 (97.6)	551 (98.0)	130 (97.7)	
OCT	14 (0.7)	2 (2.1)	1 (0.2)	6 (0.8)	4 (0.7)	1 (0.8)	
None	37 (1.8)	3 (3.1)	12 (2.5)	13 (1.7)	7 (1.2)	2 (1.5)	
Drug-eluting stent	1,893 (91.9)	84 (87.5)	448 (91.6)	721 (92.6)	513 (91.3)	127 (95.5)	0.230
Multivessel PCI	578 (28.1)	20 (20.8)	116 (23.7)	225 (28.9)	173 (30.8)	44 (33.1)	0.026
Post-PCI TIMI flow grade							0.954
0	13 (0.6)	1 (1.0)	4 (0.8)	3 (0.4)	4 (0.7)	1 (0.8)	
1	23 (1.1)	2 (2.1)	5 (1.0)	9 (1.2)	5 (0.9)	2 (1.5)	
2	162 (7.9)	8 (8.3)	45 (9.2)	62 (8.0)	38 (6.8)	9 (6.8)	
3	1,861 (90.4)	85 (88.5)	435 (89.0)	705 (90.5)	515 (91.6)	121 (91.0)	

Values are n (%).

IVUS = intravascular ultrasound; LAD = left anterior descending coronary artery; LCX = left circumflex; LMT = left main trunk; OCT = optical coherence tomography; PCI = percutaneous coronary intervention; RCA = right coronary artery; TIMI = Thrombolysis In Myocardial Infarction; other abbreviation as in Table 1.

**Figure 2:**
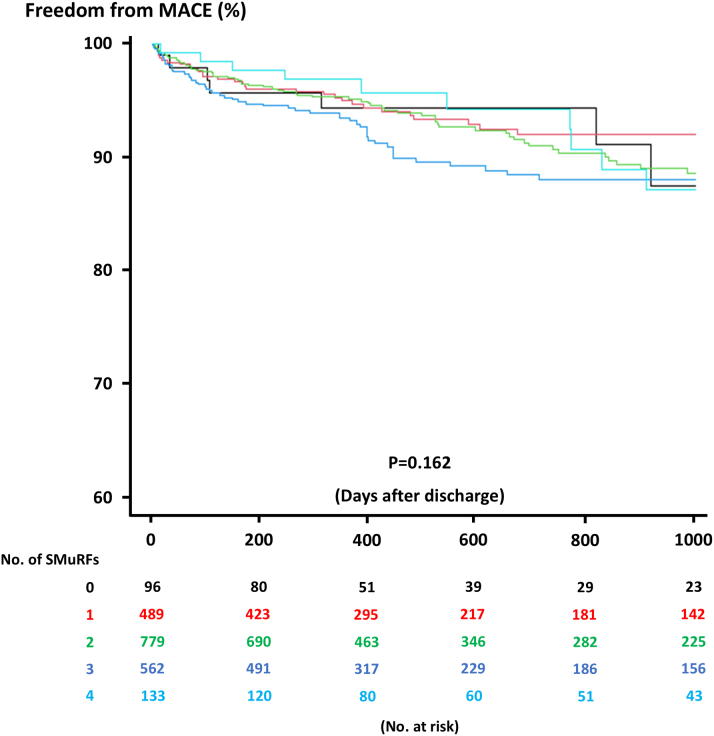
Probability Free From MACE The MACE risk after discharge did not differ significantly among the 5 groups. MACE = major adverse cardiovascular events; other abbreviation as in Figure 1.

**Figure 3:**
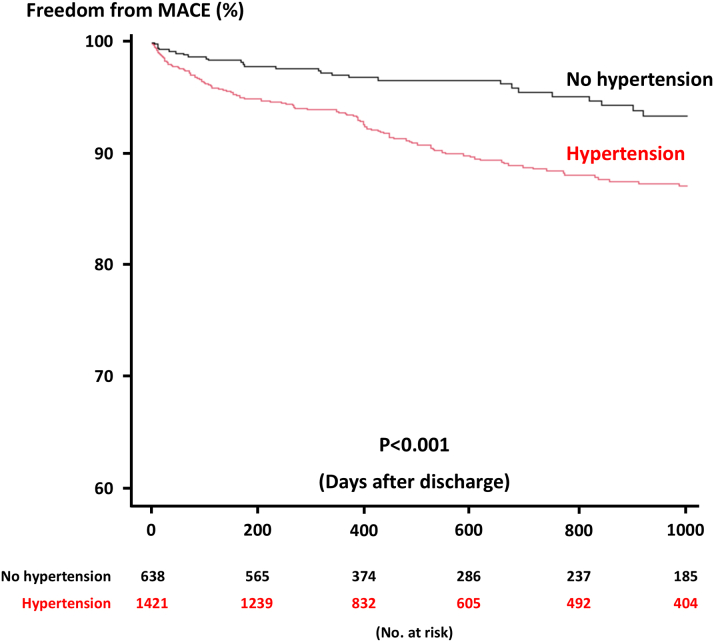
Probability Free From MACE in Patients With and Without Hypertension The MACE risk after discharge was significantly higher in patients with hypertension than in those without. Abbreviation as in Figure 1.

During a median follow-up of 538 [349-1316] days after discharge, an MACE occurred in 210 of 2,059 (10.2%) patients (Table 3). The incidence of the overall MACE did not differ significantly among the 5 groups (Table 3, Figure 2), whereas the risk of recurrent AMI increased in a stepwise manner with the increasing number of SMuRFs (Table 3). The multivariable analysis identified hypertension, previous heart failure and myocardial infarction, impaired renal function and left ventricular ejection fraction, and anemia as factors associated with MACEs during the follow-up after discharge (Table 4). Kaplan-Meier analysis showed an increased risk of MACE in patients with hypertension than in those without (Figure 3). Among the 4 SMuRFs, the combination of hypertension with diabetes remained statistically significant in the multivariable analysis for MACE, whereas other combinations were not significantly associated with the primary endpoint (Table 5). The overall results were consistent between both sexes (Supplemental Tables 2 to 5) and in the sensitivity analysis (Supplemental Tables 6 and 7).

**Table 3:** Clinical Outcomes After Discharge

	All (N = 2,059)	Number of SMuRFs					*P* Value
		0 (n = 96)	1 (n = 489)	2 (n = 779)	3 (n = 562)	4 (n = 133)	
Follow-up, d	538 [349-1,316]	468 [326-1,237]	555 [350-1,339]	560 [352-1,306]	500 [344-1,280]	546 [354-1,634]	0.466
MACE	210 (10.2) (12.7 [10.8-14.8])	9 (9.4) (12.6 [5.5-27.5])	42 (8.6) (10.1 [7.0-12.5])	75 (9.6) (12.6 [9.8-16.2])	70 (12.5) (14.4 [11.0-18.8])	14 (10.5) (15.1 [8.3-26.5])	0.101
CV death	48 (2.3) (2.6 [1.8-3.8])	3 (3.1) (2.2 [0.6-8.5])	11 (2.2) (2.5 [1.2-5.3])	20 (2.6) (3.3 [1.9-5.8])	11 (2.0) (1.8 [0.8-3.9])	3 (2.3) (2.8 [1.1-5.0])	0.508
HF rehospitalization	88 (4.3) (5.3 [4.1-6.7])	5 (5.2) (7.3 [2.3-21.3])	13 (2.7) (2.3 [1.2-4.2])	34 (4.4) (5.9 [4.2-8.4])	28 (5.0) (5.6 [3.5-8.8])	8 (6.0) (9.2 [4.2-19.4])	0.114
Recurrent AMI	67 (3.3) (3.9 [2.9-5.3])	1 (1.0) (0)	11 (2.2) (2.5 [1.2-5.4])	23 (3.0) (3.1 [1.8-5.4])	25 (4.4) (5.6 [3.4-9.1])	7 (5.3) (8.5 [3.8-18.7])	0.008
Ischemic stroke	42 (2.0) (2.8 [1.9-4.0])	2 (2.1) (6.9 [1.8-25.1])	13 (2.7) (3.3 [1.7-6.4])	11 (1.4) (2.2 [1.1-4.4])	16 (2.8) (3.3 [1.8-5.9])	0 (0) (0)	0.484
All-cause death	135 (6.6) (6.5 [5.2-8.2])	10 (10.4) (6.5 [3.0-13.9])	34 (7.0) (7.3 [4.7-11.1])	49 (6.3) (6.5 [4.5-9.5])	34 (6.0) (5.5 [3.6-8.3])	8 (6.0) (7.8 [3.4-17.4])	0.159
Non-CV death	87 (4.2) (4.0 [3.0-5.3])	7 (7.3) (4.4 [1.7-11.3])	23 (4.7) (4.9 [2.9-8.3])	29 (3.7) (3.4 [2.0-5.6])	23 (4.1) (3.7 [2.2-6.3])	5 (3.8) (5.2 [1.9-14.0])	0.206

Values represent the crude number and percentage of each event and the incidence at 3 years on Kaplan-Meier estimates [95% CIs], or median [IQR] for the follow-up period.

AMI = acute myocardial infarction; CV = cardiovascular; MACE = major adverse cardiovascular events; other abbreviations as in Table 1.

**Table 4:** Cox Proportional Hazards Analysis for MACE After Discharge

	Univariable		Multivariable	
	HR (95% CI)	*P* Value	HR (95% CI)	*P* Value
Age, y	1.028 (1.015-1.041)	<0.001	1.008 (0.992-1.024)	0.318
Men	0.835 (0.609-1.144)	0.261	1.034 (0.722-1.481)	0.856
BMI, kg/m^2^	0.964 (0.928-1.000)	0.050	0.994 (0.954-1.035)	0.755
SMuRFs				
Hypertension	2.131 (1.494-3.040)	<0.001	1.629 (1.117-2.376)	0.011
Diabetes	1.431 (1.089-1.880)	0.010	1.092 (0.811-1.471)	0.562
Dyslipidemia	0.892 (0.672-1.183)	0.426	0.919 (0.680-1.244)	0.585
Current smoker	0.672 (0.497-0.910)	0.010	0.931 (0.657-1.318)	0.685
Previous HF	5.048 (2.981-8.549)	<0.001	2.319 (1.284-4.189)	0.005
Previous MI	2.134 (1.433-3.177)	<0.001	1.616 (1.037-2.518)	0.034
LVEF, %	0.976 (0.965-0.987)	<0.001	0.987 (0.976-0.999)	0.032
eGFR, mL/min/1.73 m^2^	0.975 (0.969-0.981)	<0.001	0.987 (0.980-0.993)	<0.001
Hemoglobin, g/dL	0.824 (0.779-0.872)	<0.001	0.889 (0.827-0.957)	0.002
STEMI	1.177 (0.872-1.588)	0.287	1.401 (1.004-1.956)	0.048
Cardiogenic shock	2.009 (1.397-2.890)	<0.001	1.328 (0.873-2.019)	0.185
3-vessel disease	1.596 (1.184-2.152)	<0.001	1.358 (0.989-1.865)	0.059

Abbreviations as in Tables 1 and 3.

**Table 5:** Cox Proportional Hazards Analysis for MACE

	Multivariable	
	HR (95% CI)	*P* Value
Model 1		
Hypertension and diabetes	1.282 (1.029-1.598)	0.027
Model 2		
Hypertension and current smoker	1.211 (0.947-1.548)	0.127
Model 3		
Hypertension and dyslipidemia	1.170 (0.933-1.467)	0.175
Model 4		
Hypertension, diabetes, dyslipidemia, and current smoker	1.100 (0.942-1.283)	0.228

Combinations of SMuRFs are adjusted with other variables listed in Table 4.

Abbreviations as in Tables 1 and 3.

## Discussion

In this multicenter registry study of 2,059 patients with AMI undergoing PCI, the lack of SMuRFs was observed in approximately 5%, whereas >70% of patients had multiple SMuRFs. Although the risk of recurrent AMI was correlated with the burden of SMuRFs, the overall incidence of MACE after discharge did not differ significantly according to the cumulative number of SMuRFs (Central Illustration). Among the 4 SMuRFs, hypertension was a factor significantly associated with MACE after discharge.

**Central Illustration:**
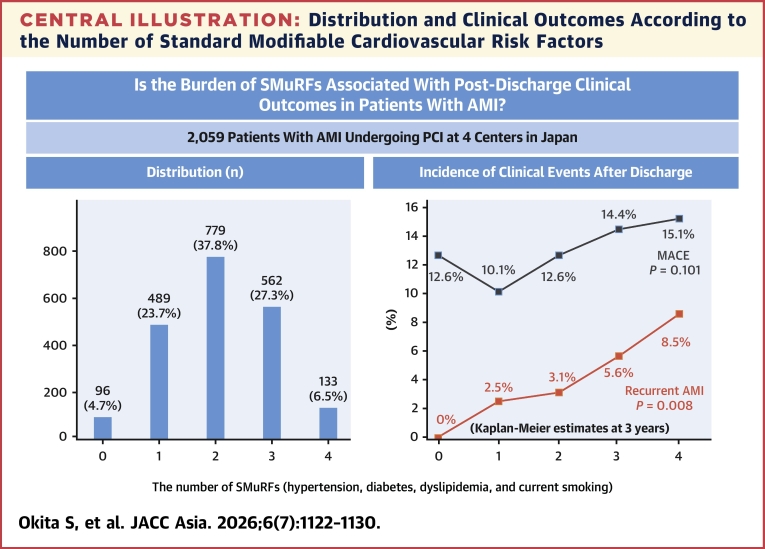
Distribution and Clinical Outcomes According to the Number of Standard Modifiable Cardiovascular Risk Factors In the present multicenter registry study in Japan, the association between the burden of SMuRFs and the risk of ischemic events after discharge was evaluated in patients with AMI. The number of SMuRFs was normally distributed, with 4.7% of patients having no SMuRFs (left figure). The increase in the number of SMuRFs was not directly related to an increased MACE risk after discharge, whereas it was significantly associated with the higher incidence of recurrent AMI. AMI = acute myocardial infarction; MACE = major adverse cardiovascular events; PCI = percutaneous coronary intervention; SMuRF = standard modifiable cardiovascular risk factor.

### Standard risk factors in AMI

SMuRFs, such as hypertension, diabetes, dyslipidemia, and current smoking, account for a considerable proportion of the risk of atherosclerotic cardiovascular disease, including AMI.^1^ However, even with no SMuRFs, patients can develop AMI due to nontraditional risk factors, such as inflammatory diseases, elevated levels of lipoprotein(a), malignancy, infectious diseases, poor social determinants of health, environmental pollution, mental distress, and unhealthy sleep.^33^ Although mechanistically unclear,^6^ the lack of SMuRFs is reportedly associated with poor clinical outcomes after AMI, particularly during hospitalization.^7^, ^8^, ^9^, ^10^, ^11^, ^12^ Following the acute phase, the prognostic impact of no SMuRFs after discharge in patients with AMI has not been established yet. A large-scale registry study from Japan showed that the lack of SMuRFs was significantly associated with an increased mortality risk during the median follow-up period of 5.6 years in patients with STEMI but not in those with NSTEMI.^13^ Even in patients with STEMI, the difference in all-cause death was mainly driven by rapidly increasing mortality immediately after the index event in SMuRF-less patients, whereas in those with chronic coronary artery disease, the survival curves showed a progressive time-dependent divergence in the Japanese registry.^13^ In addition, conflicting results have been reported in this context.^8^^,^^14^, ^15^, ^16^, ^17^ Thus, the prognostic impact of the lack of SMuRFs after discharge in patients with AMI remains uncertain. Beyond dichotomous evaluation with and without being SMuRF-less, the relationship between the cumulative number of SMuRFs and clinical outcomes after AMI is even unclear.

### Burden of risk factors and long-term outcomes

Intuitively, the accumulation of risks leads to cardiovascular events.^5^ The present study showed that the number of SMuRFs, from 0 to 4, was normally distributed, as was illustrated in previous reports.^34^ Patients having all 4 SMuRFs were not common in this study (ie, 6.5%), which is in line with previous investigations showing the prevalence of 0.2% to 8.7%.^34^^,^^35^ Among patients with chronic coronary artery disease, the CLARIFY registry demonstrated the linear dose-response relationship between the burden of SMuRFs and the risk of death or AMI,^18^ whereas in patients with AMI, the relation remained to be established. Even though the incidence of the primary endpoint did not differ significantly among the 5 groups, the risk of recurrent AMI was higher in patients with a greater burden of SMuRFs in our study. The 5-year risk of death or AMI was 5.4%, 6.0%, 7.3%, 9.0%, and 9.7% in the CLARIFY registry,^18^ and the incidence of recurrent AMI was 1.0%, 2.2%, 3.0%, 4.4%, and 5.3% during the median follow-up period of 538 days in the present study in patients having 0, 1, 2, 3, and 4 SMuRFs. No significant association between the overall MACE risk and burden of SMuRFs may be attributable to different clinical characteristics and presentations of patients with acute and chronic coronary syndromes.^36^ Nonetheless, we believe that the present study results reinforce the importance of secondary prevention after AMI by targeting SMuRFs.^2^, ^3^, ^4^ Interestingly, hypertension was identified as a predictor of MACE during follow-up after AMI among the traditional risks. The finding may fit the subanalysis results of the ISCHEMIA (International Study of Comparative Health Effectiveness With Medical and Invasive Approaches) trial, showing that earlier achievement and maintenance of blood pressure targets were associated with lower long-term cardiovascular risks.^37^ In addition, the presence of diabetes on top of hypertension remained significant for predicting MACEs. Therefore, comprehensive risk management, mainly against hypertension and diabetes, could convey better secondary prevention after AMI, although further studies are warranted to evaluate if the risk-based approach can improve clinical outcomes in patients with AMI.

### Study limitations

Because this was a retrospective, observational study, a cause-and-effect relationship cannot be established. Most of the study population were men, and women were underrepresented. The lack of SMuRFs is reportedly associated with an increased in-hospital mortality in patients with AMI.^7^, ^8^, ^9^, ^10^, ^11^, ^12^ Although unestablished, underreporting of the SMuRFs in severely ill patients may be a possible mechanism.^6^ To reduce the bias and to focus on long-term clinical outcomes after discharge, we excluded patients who died during hospitalization for the index AMI event, potentially resulting in a survival bias. For individual SMuRFs, the presence or absence was identified, whereas the dose-response relationship between risk factors (eg, blood pressure levels and glycemic control) and clinical outcomes was not evaluated in the present study. Although the cumulative exposure (eg, duration and severity) to SMuRFs is associated with an increased cardiovascular risk,^38^ the information was also unavailable. In addition, detailed information on vital signs, blood examination, electrocardiography (eg, localization and QRS duration), echocardiography, angiography and intracoronary imaging (eg, lesion severity and morphology), and pharmacological treatment during the follow-up period was lacking. The definitions of SMuRFs and prevalence of SMuRF-less patients in AMI vary widely among studies and regions,^6^ whereas the present study predominantly included East Asian patients. Thus, whether our findings can be extrapolated to other populations remains uncertain.

## Conclusions

In patients with AMI undergoing PCI, the presence of multiple SMuRFs, including hypertension, diabetes, dyslipidemia, and current smoking, was common. The burden of SMuRFs was not significantly associated with an increased overall risk of MACEs, whereas the cumulative incidence of recurrent AMI was linearly and progressively correlated with the increase in the number of SMuRFs. Among the 4 SMuRFs, the presence of hypertension was particularly associated with worse outcomes. Our findings suggest the complex interplay of traditional cardiovascular risk factors with ischemic outcomes and the potential that a cumulative risk-based therapeutic intervention may improve outcomes in patients with AMI.

## Funding Support and Author Disclosures

This work was supported by grants from Takeda Science Foundation. The authors have reported that they have no relationships relevant to the contents of this paper to disclose.
