# Supplementary material for: Incremental Impact of Standard Modifiable Cardiovascular Risk Factors on Long-Term Outcomes After Acute Myocardial Infarction
Source: JACC Asia. 2026 May 6;6(7):1122–30. doi: 10.1016/j.jacasi.2026.03.030 (PMC13350681; doi:10.1016/j.jacasi.2026.03.030)
Supplement: Supplemental Tables 1-7 [file mmc1.docx]

**Supplemental Table 1 Distribution of SMuRF combinations**

| Variable | All  (n=2,059) |
| --- | --- |
|  |  |
| 0 SMuRFs (n=96) |  |
| None | 96 (4.7) |
| 1 SMuRF (n=489) |  |
| Hypertension | 208 (10.1) |
| Diabetes | 36 (1.7) |
| Dyslipidemia | 173 (8.4) |
| Smoking | 72 (3.5) |
| 2 SMuRFs (n=779) |  |
| Hypertension and diabetes | 97 (4.7) |
| Hypertension and dyslipidemia | 363 (17.6) |
| Hypertension and smoking | 114 (5.5) |
| Diabetes and dyslipidemia | 58 (2.8) |
| Diabetes and smoking | 29 (1.4) |
| Dyslipidemia and smoking | 118 (5.7) |
| 3 SMuRFs (n=562) |  |
| Hypertension, diabetes, and dyslipidemia | 277 (13.5) |
| Hypertension, diabetes, and smoking | 50 (2.4) |
| Hypertension, dyslipidemia, and smoking | 179 (8.7) |
| Diabetes, dyslipidemia, and smoking | 56 (2.7) |
| 4 SMuRFs (n=133) |  |
| Hypertension, diabetes, dyslipidemia, and smoking | 133 (6.5) |

Values are n (%).

SMuRF = standard modifiable cardiovascular risk factor.

**Supplemental Table 2 Baseline Characteristics According to the Number of SMuRFs in Men**

| Variable | All  (n=1,597) | The number of SMuRFs | | | | | P value |
| --- | --- | --- | --- | --- | --- | --- | --- |
|  |  | 0  (n=72) | 1  (n=365) | 2  (n=599) | 3  (n=437) | 4  (n=124) |  |
| Age, years | 65.7±12.3 | 67.3±14.0 | 67.9±12.1 | 66.1±12.3 | 64.1±12.0 | 61.8±11.4 | <0.001 |
| Men | 1,597 (100) | 72 (100) | 365 (100) | 599 (100) | 437 (100) | 124 (100) | NA |
| BMI, kg/m^2^ | 24.7±3.7 | 23.0±3.2 | 24.0±3.5 | 24.6±3.3 | 25.1±3.9 | 26.8±4.4 | <0.001 |
| Hypertension | 1,093 (68.4) | 0 (0) | 154 (42.2) | 425 (71.0) | 390 (89.2) | 124 (100) | <0.001 |
| Diabetes | 564 (35.3) | 0 (0) | 28 (7.7) | 138 (23.0) | 274 (62.7) | 124 (100) | <0.001 |
| Dyslipidemia | 1,024 (64.1) | 0 (0) | 113 (31.0) | 399 (66.6) | 388 (88.8) | 124 (100) | <0.001 |
| Current smoker | 689 (43.1) | 0 (0) | 70 (19.2) | 236 (39.4) | 259 (59.3) | 124 (100) | <0.001 |
| Active cancer | 71 (4.4) | 7 (9.7) | 21 (5.8) | 20 (3.3) | 17 (3.9) | 6 (4.8) | 0.082 |
| Previous HF | 31 (1.9) | 1 (1.4) | 6 (1.6) | 13 (2.2) | 9 (2.1) | 2 (1.6) | 0.968 |
| Previous MI | 135 (8.5) | 2 (2.8) | 16 (4.4) | 51 (8.5) | 55 (12.6) | 11 (8.9) | <0.001 |
| Atrial fibrillation | 82 (5.1) | 6 (8.3) | 25 (6.8) | 31 (5.2) | 17 (3.9) | 3 (2.4) | 0.137 |
| MCS device |  |  |  |  |  |  |  |
| IABP | 106 (6.6) | 5 (6.9) | 23 (6.3) | 37 (6.2) | 34 (7.8) | 7 (5.6) | 0.844 |
| VA-ECMO | 29 (1.8) | 3 (4.2) | 8 (2.2) | 9 (1.5) | 8 (1.8) | 1 (0.8) | 0.469 |
| Echocardiographic data |  |  |  |  |  |  |  |
| LVEF, % | 48.7±11.8 | 47.1±12.6 | 49.9±12.5 | 48.0±12.0 | 48.5±10.6 | 49.9±12.1 | 0.073 |
| Electrocardiographic data |  |  |  |  |  |  |  |
| STEMI | 1,116 (69.9) | 54 (75.0) | 249 (68.2) | 421 (70.3) | 307 (70.3) | 85 (68.5) | 0.817 |
| NSTEMI | 481 (30.1) | 18 (25.0) | 116 (31.8) | 178 (29.7) | 130 (29.7) | 39 (31.5) | 0.817 |
| ST-segment deviation | 157 (32.6) | 5 (27.8) | 43 (37.1) | 59 (33.1) | 38 (29.2) | 12 (30.8) | 0.736 |
| eGFR, mL/min/1.73 m^2^ | 66.4±23.2 | 66.8±16.8 | 65.0±21.8 | 65.6±22.3 | 67.4±25.3 | 70.9±26.6 | 0.110 |
| Hemoglobin, g/dL | 14.4±2.0 | 13.5±2.1 | 14.1±2.0 | 14.5±1.9 | 14.5±2.1 | 15.2±1.9 | <0.001 |
| HbA1c, % | 6.4±1.3 | 5.7±0.4 | 5.8±0.7 | 6.1±1.1 | 6.9±1.7 | 7.7±1.5 | <0.001 |
| LDL-C, mg/dL | 122.7±40.0 | 110.1±34.8 | 120.7±38.1 | 125.6±38.8 | 122.8±41.8 | 121.8±40.7 | 0.023 |
| Clinical presentation |  |  |  |  |  |  |  |
| Cardiogenic shock | 151 (9.5) | 5 (6.9) | 39 (10.7) | 56 (9.3) | 42 (9.6) | 9 (7.3) | 0.755 |
| Cardiac arrest | 108 (6.8) | 4 (5.6) | 23 (6.3) | 47 (7.8) | 29 (6.6) | 5 (4.0) | 0.578 |
| Procedural findings |  |  |  |  |  |  |  |
| Culprit vessel |  |  |  |  |  |  | 0.156 |
| RCA | 511 (32.0) | 25 (34.7) | 99 (27.1) | 194 (32.4) | 151 (34.6) | 42 (33.9) |  |
| LAD/LMT | 798 (50.0) | 37 (51.4) | 208 (57.0) | 291 (48.6) | 203 (46.6) | 59 (47.6) |  |
| LCX | 245 (15.4) | 9 (12.5) | 52 (14.2) | 101 (16.9) | 64 (14.7) | 19 (15.3) |  |
| 3-vessel disease | 294 (18.4) | 7 (9.7) | 51 (14.0) | 108 (18.0) | 103 (23.6) | 25 (20.2) | 0.002 |
| Access site |  |  |  |  |  |  | 0.648 |
| Radial artery | 1,492 (93.4) | 68 (94.4) | 337 (92.3) | 558 (93.2) | 410 (93.8) | 119 (96.0) |  |
| Femoral artery | 90 (5.6) | 4 (5.6) | 25 (6.8) | 32 (5.3) | 24 (5.5) | 5 (4.0) |  |
| Others | 15 (0.9) | 0 (0) | 3 (0.8) | 9 (1.5) | 3 (0.7) | 0 (0) |  |
| Intracoronary imaging |  |  |  |  |  |  | 0.409 |
| IVUS | 1,562 (97.8) | 69 (95.8) | 356 (97.5) | 585 (97.7) | 431 (98.6) | 121 (97.6) |  |
| OCT | 11 (0.7) | 2 (2.8) | 1 (0.3) | 5 (0.8) | 2 (0.5) | 1 (0.8) |  |
| None | 24 (1.5) | 1 (1.4) | 8 (2.2) | 9 (1.5) | 4 (0.9) | 2 (1.6) |  |
| Drug-eluting stent | 1,467 (91.9) | 63 (87.5) | 336 (92.1) | 551 (92.0) | 399 (91.3) | 118 (95.2) | 0.427 |
| Multivessel PCI | 460 (28.8) | 17 (23.6) | 95 (26.0) | 174 (29.0) | 132 (30.2) | 42 (33.9) | 0.366 |
| Post-PCI TIMI flow grade |  |  |  |  |  |  | 0.786 |
| 0 | 8 (0.5) | 1 (1.4) | 1 (0.3) | 2 (0.3) | 3 (0.7) | 1 (0.8) |  |
| 1 | 20 (1.3) | 2 (2.8) | 5 (1.4) | 6 (1.0) | 5 (1.1) | 2 (1.6) |  |
| 2 | 125 (7.8) | 4 (5.6) | 37 (10.1) | 44 (7.3) | 31 (7.1) | 9 (7.3) |  |
| 3 | 1444 (90.4) | 65 (90.3) | 322 (88.2) | 547 (91.3) | 398 (91.1) | 112 (90.3) |  |
| Medications at discharge |  |  |  |  |  |  |  |
| Aspirin | 1,483 (92.9) | 67 (93.1) | 334 (91.5) | 552 (92.2) | 410 (93.8) | 120 (96.8) | 0.294 |
| P2Y12 inhibitor | 1,542 (96.6) | 65 (90.3) | 356 (97.5) | 576 (96.2) | 425 (97.3) | 120 (96.8) | 0.032 |
| Oral anticoagulation | 211 (13.2) | 7 (9.7) | 66 (18.1) | 81 (13.5) | 52 (11.9) | 5 (4.0) | 0.001 |
| Statin | 1,487 (93.1) | 65 (90.3) | 328 (89.9) | 564 (94.2) | 411 (94.1) | 119 (96.0) | 0.039 |
| SGLT-2 inhibitor | 160 (10.0) | 1 (1.4) | 11 (3.0) | 31 (5.2) | 78 (17.8) | 39 (31.5) | <0.001 |
| Insulin | 51 (3.2) | 0 (0) | 3 (0.8) | 17 (2.8) | 26 (5.9) | 5 (4.0) | <0.001 |
| Calcium channel blocker | 302 (18.9) | 4 (5.6) | 56 (15.3) | 108 (18.0) | 105 (24.0) | 29 (23.4) | <0.001 |
| ACE-I, ARB, or ARNI | 1,266 (79.3) | 51 (70.8) | 273 (74.8) | 473 (79.0) | 365 (83.5) | 104 (83.9) | 0.007 |
| β-blocker | 1,216 (76.1) | 56 (77.8) | 268 (73.4) | 450 (75.1) | 339 (77.6) | 103 (83.1) | 0.223 |
| MRA | 251 (15.7) | 13 (18.1) | 57 (15.6) | 91 (15.2) | 72 (16.5) | 18 (14.5) | 0.945 |
| Diuretic | 269 (16.8) | 14 (19.4) | 58 (15.9) | 100 (16.7) | 80 (18.3) | 17 (13.7) | 0.712 |

Values are mean ± SD or n (%).

ACE-I = angiotensin-converting enzyme inhibitor; ARB = angiotensin II receptor blocker; ARNI = angiotensin receptor neprilysin inhibitor; BMI = body mass index; eGFR = estimated glomerular filtration rate; HbA1c = glycated hemoglobin; HF = heart failure; IABP = intra-aortic balloon pump; IVUS = intravascular ultrasound; LAD = left anterior descending coronary artery; LCX = left circumflex; LDL-C = low-density lipoprotein cholesterol; LMT = left main trunk; LVEF = left ventricular ejection fraction; MCS = mechanical circulatory support; MI = myocardial infarction; MRA = mineralocorticoid receptor antagonist; NA = not applicable; NSTEMI = non-ST-segment elevation myocardial infarction; OCT = optical coherence tomography; PCI = percutaneous coronary intervention; RCA = right coronary artery; SGLT-2 = sodium-glucose cotransporter 2; SMuRF = standard modifiable cardiovascular risk factor; STEMI = ST-segment elevation myocardial infarction; TIMI = Thrombolysis In Myocardial Infarction; VA-ECMO = veno-arterial extracorporeal membrane oxygenation.

**Supplemental Table 3 Clinical Outcomes After Discharge in Men**

| Variable | All  (n=1,597) | The number of SMuRFs | | | | | P value |
| --- | --- | --- | --- | --- | --- | --- | --- |
|  |  | 0  (n=72) | 1  (n=365) | 2  (n=599) | 3  (n=437) | 4  (n=124) |  |
| Follow-up, days | 555  [355-1,350] | 554  [326-1,443] | 585  [364-1,431] | 579  [355-1,343] | 500  [349-1,142] | 582  [348-1,642] | 0.167 |
| MACE | 159 (10.0%)  (12.0 [10.0-14.5]) | 7 (9.7%)  (7.5 [3.2-17.2]) | 30 (8.2%)  (9.4 [6.0-14.4]) | 60 (10.0%)  (13.2 [9.9-17.5]) | 48 (11.0%)  (12.1 [8.5-17.2]) | 14 (11.3%)  (15.8 [8.7-27.7]) | 0.159 |
| CV death | 39 (2.4%)  (2.9 [1.9-4.3]) | 3 (4.2%)  (2.9 [0.7-11.0]) | 6 (1.6%)  (2.1 [0.7-5.8]) | 18 (3.0%)  (3.8 [2.0-6.9]) | 9 (2.1%)  (2.1 [0.9-4.7]) | 3 (2.4%)  (2.9 [0.7-12.3]) | 0.823 |
| HF rehospitalization | 62 (3.9%)  (4.7 [3.5-6.2]) | 4 (5.6%)  (4.7 [1.5-14.1]) | 8 (2.2%)  (1.9 [0.8-4.2]) | 27 (4.5%)  (5.9 [4.0-8.6]) | 15 (3.4%)  (3.6 [1.9-7.1]) | 8 (6.5%)  (9.6 [4.4-20.3]) | 0.248 |
| Recurrent AMI | 50 (3.1%)  (4.0 [2.8-5.6]) | 1 (1.4%)  (0) | 9 (2.5%)  (2.9 [1.3-6.5]) | 17 (2.8%)  (3.3 [1.8-6.3]) | 16 (3.7%)  (4.9 [2.5-9.2]) | 7 (5.6%)  (8.9 [3.9-19.5]) | 0.043 |
| Ischemic stroke | 31 (1.9%)  (2.5 [1.6-3.8]) | 0 (0%)  (0) | 8 (2.2%)  (2.5 [1.1-6.1]) | 9 (1.5%)  (2.4 [1.1-5.2]) | 14 (3.2%)  (3.6 [1.9-6.8]) | 0 (0%)  (0) | 0.586 |
| All-cause death | 108 (6.8%)  (6.9 [5.4-8.8]) | 8 (11.1%)  (5.8 [2.2-14.6]) | 23 (6.3%)  (6.6 [3.9-11.0]) | 44 (7.3%)  (7.3 [4.8-11.0]) | 25 (5.7%)  (6.0 [3.7-9.6]) | 8 (6.5%)  (8.2 [3.6-18.1]) | 0.375 |
| Non-CV death | 69 (4.3%)  (4.1 [3.0-5.7]) | 5 (6.9%)  (3.0 [0.7-11.3]) | 17 (4.7%)  (4.6 [2.5-8.4]) | 26 (4.3%)  (3.7 [2.1-6.5]) | 16 (3.7%)  (4.0 [2.2-7.2]) | 5 (4.0%)  (5.4 [2.0-14.6]) | 0.347 |

Values represent the crude number and percentage of each event and the incidence at 3 years on Kaplan-Meier estimates [95% confidence intervals], or median [interquartile range] for the follow-up period.

AMI = acute myocardial infarction; CV = cardiovascular; HF = heart failure; MACE = major adverse cardiovascular events; SMuRF = standard modifiable cardiovascular risk factor.

**Supplemental Table 4 Baseline Characteristics According to the Number of SMuRFs in Women**

| Variable | All  (n=462) | The number of SMuRFs | | | | | P value |
| --- | --- | --- | --- | --- | --- | --- | --- |
|  |  | 0  (n=24) | 1  (n=124) | 2  (n=180) | 3  (n=125) | 4  (n=9) |  |
| Age, years | 73.1±10.9 | 76.2±14.4 | 72.9±11.1 | 74.9±10.0 | 71.2±10.3 | 57.4±5.7 | <0.001 |
| Men | 0 (0) | 0 (0) | 0 (0) | 0 (0) | 0 (0) | 0 (0) | NA |
| BMI, kg/m^2^ | 23.5±4.2 | 22.9±3.9 | 22.1±3.5 | 23.2±3.7 | 24.9±4.6 | 28.2±6.7 | <0.001 |
| Hypertension | 328 (71.0) | 0 (0) | 54 (43.5) | 149 (82.8) | 116 (92.8) | 9 (100) | <0.001 |
| Diabetes | 172 (37.2) | 0 (0) | 8 (6.5) | 46 (25.6) | 109 (87.2) | 9 (100) | <0.001 |
| Dyslipidemia | 333 (72.1) | 0 (0) | 60 (48.4) | 140 (77.8) | 124 (99.2) | 9 (100) | <0.001 |
| Current smoker | 62 (13.4) | 0 (0) | 2 (1.6) | 25 (13.9) | 26 (20.8) | 9 (100) | <0.001 |
| Active cancer | 15 (3.2) | 3 (12.5) | 7 (5.6) | 4 (2.2) | 1 (0.8) | 0 (0) | 0.017 |
| Previous HF | 9 (1.9) | 0 (0) | 2 (1.6) | 2 (1.1) | 5 (4.0) | 0 (0) | 0.387 |
| Previous MI | 20 (4.3) | 0 (0) | 4 (3.2) | 8 (4.4) | 8 (6.4) | 0 (0) | 0.532 |
| Atrial fibrillation | 30 (6.5) | 2 (8.3) | 13 (10.5) | 7 (3.9) | 8 (6.4) | 0 (0) | 0.197 |
| MCS device |  |  |  |  |  |  |  |
| IABP | 29 (6.3) | 1 (4.2) | 7 (5.6) | 11 (6.1) | 10 (8.0) | 0 (0) | 0.825 |
| VA-ECMO | 6 (1.3) | 0 (0) | 1 (0.8) | 3 (1.7) | 2 (1.6) | 0 (0) | 0.918 |
| Echocardiographic data |  |  |  |  |  |  |  |
| LVEF, % | 50.3±12.2 | 55.6±11.7 | 50.9±13.0 | 48.8±11.7 | 50.7±12.5 | 50.1±7.4 | 0.108 |
| Electrocardiographic data |  |  |  |  |  |  |  |
| STEMI | 306 (66.2) | 17 (70.8) | 85 (68.5) | 122 (67.8) | 75 (60.0) | 7 (77.8) | 0.490 |
| NSTEMI | 156 (33.8) | 7 (29.2) | 39 (31.5) | 58 (32.2) | 50 (40.0) | 2 (22.2) | 0.490 |
| ST-segment deviation | 49 (31.4) | 3 (42.9) | 12 (30.8) | 16 (27.6) | 17 (34.0) | 1 (50.0) | 0.861 |
| eGFR, mL/min/1.73 m^2^ | 66.6±24.6 | 65.7±17.1 | 65.7±22.4 | 69.7±23.0 | 61.6±28.1 | 89.5±33.5 | 0.003 |
| Hemoglobin, g/dL | 12.6±1.9 | 11.8±2.5 | 12.5±1.7 | 12.9±1.7 | 12.4±2.2 | 14.2±1.0 | 0.003 |
| HbA1c, % | 6.5±1.5 | 5.6±0.3 | 5.8±0.6 | 6.3±1.1 | 7.5±1.9 | 9.5±2.5 | <0.001 |
| LDL-C, mg/dL | 125.8±45.8 | 113.7±30.7 | 130.8±46.2 | 127.3±40.8 | 119.4±53.4 | 145.7±44.8 | 0.125 |
| Clinical presentation |  |  |  |  |  |  |  |
| Cardiogenic shock | 39 (8.4) | 2 (8.3) | 8 (6.5) | 16 (8.9) | 13 (10.4) | 0 (0) | 0.711 |
| Cardiac arrest | 17 (3.7) | 3 (12.5) | 4 (3.2) | 5 (2.8) | 5 (4.0) | 0 (0) | 0.189 |
| Procedural findings |  |  |  |  |  |  |  |
| Culprit vessel |  |  |  |  |  |  | 0.856 |
| RCA | 166 (35.9) | 8 (33.3) | 43 (34.7) | 62 (34.4) | 49 (39.2) | 4 (44.4) |  |
| LAD/LMT | 216 (46.8) | 12 (50.0) | 57 (46.0) | 93 (51.7) | 50 (40.0) | 4 (44.4) |  |
| LCX | 68 (14.7) | 4 (16.7) | 20 (16.1) | 22 (12.2) | 21 (16.8) | 1 (11.1) |  |
| 3-vessel disease | 83 (18.0) | 6 (25.0) | 10 (8.1) | 28 (15.6) | 38 (30.4) | 1 (11.1) | <0.001 |
| Access site |  |  |  |  |  |  | 0.129 |
| Radial artery | 404 (87.4) | 23 (95.8) | 113 (91.1) | 161 (89.4) | 100 (80.0) | 7 (77.8) |  |
| Femoral artery | 46 (10.0) | 1 (4.2) | 8 (6.5) | 16 (8.9) | 19 (15.2) | 2 (22.2) |  |
| Others | 12 (2.6) | 0 (0) | 3 (2.4) | 3 (1.7) | 6 (4.8) | 0 (0) |  |
| Intracoronary imaging |  |  |  |  |  |  | 0.637 |
| IVUS | 446 (96.5) | 22 (91.7) | 120 (96.8) | 175 (97.2) | 120 (96.0) | 9 (100) |  |
| OCT | 3 (0.6) | 0 (0) | 0 (0) | 1 (0.6) | 2 (1.6) | 0 (0) |  |
| None | 13 (2.8) | 2 (8.3) | 4 (3.2) | 4 (2.2) | 3 (2.4) | 0 (0) |  |
| Drug-eluting stent | 426 (92.2) | 21 (87.5) | 112 (90.3) | 170 (94.4) | 114 (91.2) | 9 (100) | 0.471 |
| Multivessel PCI | 118 (25.5) | 3 (12.5) | 21 (16.9) | 51 (28.3) | 41 (32.8) | 2 (22.2) | 0.024 |
| Post-PCI TIMI flow grade |  |  |  |  |  |  | 0.347 |
| 0 | 5 (1.1) | 0 (0) | 3 (2.4) | 1 (0.6) | 1 (0.8) | 0 (0) |  |
| 1 | 3 (0.6) | 0 (0) | 0 (0) | 3 (1.7) | 0 (0) | 0 (0) |  |
| 2 | 37 (8.0) | 4 (16.7) | 8 (6.5) | 18 (10.0) | 7 (5.6) | 0 (0) |  |
| 3 | 417 (90.3) | 20 (83.3) | 113 (91.1) | 158 (87.8) | 117 (93.6) | 9 (100) |  |
| Medications at discharge |  |  |  |  |  |  |  |
| Aspirin | 420 (90.9) | 20 (83.3) | 110 (88.7) | 164 (91.1) | 117 (93.6) | 9 (100) | 0.355 |
| P2Y12 inhibitor | 436 (94.4) | 22 (91.7) | 113 (91.1) | 171 (95.0) | 121 (96.8) | 9 (100) | 0.304 |
| Oral anticoagulation | 56 (12.1) | 4 (16.7) | 20 (16.1) | 19 (10.6) | 13 (10.4) | 0 (0) | 0.362 |
| Statin | 426 (92.2) | 16 (66.7) | 108 (87.1) | 175 (97.2) | 118 (94.4) | 9 (100) | <0.001 |
| SGLT-2 inhibitor | 36 (7.8) | 0 (0) | 4 (3.2) | 10 (5.6) | 19 (15.2) | 3 (33.3) | <0.001 |
| Insulin | 29 (6.3) | 0 (0) | 3 (2.4) | 7 (3.9) | 16 (12.8) | 3 (33.3) | <0.001 |
| Calcium channel blocker | 105 (22.7) | 5 (20.8) | 18 (14.5) | 39 (21.7) | 39 (31.2) | 4 (44.4) | 0.014 |
| ACE-I, ARB, or ARNI | 351 (76.0) | 16 (66.7) | 83 (66.9) | 136 (75.6) | 108 (86.4) | 8 (88.9) | 0.005 |
| β-blocker | 321 (69.5) | 19 (79.2) | 82 (66.1) | 128 (71.1) | 87 (69.6) | 5 (55.6) | 0.597 |
| MRA | 103 (22.3) | 2 (8.3) | 25 (20.2) | 41 (22.8) | 35 (28.0) | 0 (0) | 0.092 |
| Diuretic | 109 (23.6) | 4 (16.7) | 30 (24.2) | 39 (21.7) | 36 (28.8) | 0 (0) | 0.223 |

Values are mean ± SD or n (%).

ACE-I = angiotensin-converting enzyme inhibitor; ARB = angiotensin II receptor blocker; ARNI = angiotensin receptor neprilysin inhibitor; BMI = body mass index; eGFR = estimated glomerular filtration rate; HbA1c = glycated hemoglobin; HF = heart failure; IABP = intra-aortic balloon pump; IVUS = intravascular ultrasound; LAD = left anterior descending coronary artery; LCX = left circumflex; LDL-C = low-density lipoprotein cholesterol; LMT = left main trunk; LVEF = left ventricular ejection fraction; MCS = mechanical circulatory support; MI = myocardial infarction; MRA = mineralocorticoid receptor antagonist; NA = not applicable; NSTEMI = non-ST-segment elevation myocardial infarction; OCT = optical coherence tomography; PCI = percutaneous coronary intervention; RCA = right coronary artery; SGLT-2 = sodium-glucose cotransporter 2; SMuRF = standard modifiable cardiovascular risk factor; STEMI = ST-segment elevation myocardial infarction; TIMI = Thrombolysis In Myocardial Infarction; VA-ECMO = veno-arterial extracorporeal membrane oxygenation.

**Supplemental Table 5 Clinical Outcomes After Discharge in Women**

| Variable | All  (n=462) | The number of SMuRFs | | | | | P value |
| --- | --- | --- | --- | --- | --- | --- | --- |
|  |  | 0  (n=24) | 1  (n=124) | 2  (n=180) | 3  (n=125) | 4  (n=9) |  |
| Follow-up, days | 473  [306-1,220] | 371  [335-920] | 419  [221-955] | 516  [321-1,213] | 500  [338-1,498] | 458  [358-546] | 0.129 |
| MACE | 51 (11.0%)  (15.0 [11.2-20.0]) | 2 (8.3%)  (31.4 [8.8-78.7]) | 12 (9.7%)  (11.8 [6.4-21.1]) | 15 (8.3%)  (10.8 [6.1-18.5]) | 22 (17.6%)  (22.1 [14.6-32.8]) | 0 (0%)  (0) | 0.244 |
| CV death | 9 (1.9%)  (1.8 [0.8-3.7]) | 0 (0%)  (0) | 5 (4.0%)  (3.5 [1.3-9.0]) | 2 (1.1%)  (1.6 [0.4-6.2]) | 2 (1.6%)  (0.8 [0.1-5.6]) | 0 (0%)  (0) | 0.284 |
| HF rehospitalization | 26 (5.6%)  (7.6 [4.8-11.8]) | 1 (4.2%)  (16.7 [2.5-72.7]) | 5 (4.0%)  (3.5 [1.3-9.0]) | 7 (3.9%)  (6.0 [2.6-13.6]) | 13 (10.4%)  (12.3 [6.7-22.0]) | 0 (0%)  (0) | 0.159 |
| Recurrent AMI | 17 (3.7%)  (3.6 [2.0-6.1]) | 0 (0%)  (0) | 2 (1.6%)  (0.9 [0.1-6.1]) | 6 (3.3%)  (2.3 [0.9-6.1]) | 9 (7.2%)  (8.4 [4.2-16.4]) | 0 (0%)  (0) | 0.050 |
| Ischemic stroke | 11 (2.4%)  (3.9 [2.0-7.5]) | 2 (8.3%)  (31.4 [8.8-78.7]) | 5 (4.0%)  (5.9 [2.1-15.6]) | 2 (1.1%)  (1.5 [0.4-6.2]) | 2 (1.6%)  (2.2 [0.5-9.1]) | 0 (0%)  (0) | 0.019 |
| All-cause death | 27 (5.8%)  (5.2 [3.3-8.4]) | 2 (8.3%)  (8.9 [2.3-31.2]) | 11 (8.9%)  (9.3 [4.2-20.2]) | 5 (2.8%)  (3.7 [1.5-8.8]) | 9 (7.2%)  (3.7 [1.4-9.8]) | 0 (0%)  (0) | 0.192 |
| Non-CV death | 18 (3.9%)  (3.6 [1.9-6.6]) | 2 (8.3%)  (8.9 [2.3-31.2]) | 6 (4.8%)  (6.1 [1.9-18.5]) | 3 (1.7%)  (2.2 [0.7-6.8]) | 7 (5.6%)  (2.9 [0.9-9.0]) | 0 (0%)  (0) | 0.401 |

Values represent the crude number and percentage of each event and the incidence at 3 years on Kaplan-Meier estimates [95% confidence intervals], or median [interquartile range] for the follow-up period.

AMI = acute myocardial infarction; CV = cardiovascular; HF = heart failure; MACE = major adverse cardiovascular events; SMuRF = standard modifiable cardiovascular risk factor.

**Supplemental Table 6 Cox Proportional Hazards Analysis for MACE After Discharge**

| Variable | Univariable | |  | Multivariable | |
| --- | --- | --- | --- | --- | --- |
|  | HR (95% CI) | P value |  | HR (95% CI) | P value |
| Age, years | 1.028 (1.015-1.041) | <0.001 |  | 1.009 (0.994-1.025) | 0.252 |
| SMuRFs |  |  |  |  |  |
| Hypertension | 2.131 (1.494-3.040) | <0.001 |  | 1.605 (1.100-2.340) | 0.014 |
| Diabetes | 1.431 (1.089-1.880) | 0.010 |  | 1.075 (0.797-1.449) | 0.636 |
| Dyslipidemia | 0.892 (0.672-1.183) | 0.426 |  | 0.920 (0.680-1.243) | 0.585 |
| Current smoker | 0.672 (0.497-0.910) | 0.010 |  | 0.905 (0.636-1.288) | 0.581 |
| Previous HF | 5.048 (2.981-8.549) | <0.001 |  | 2.264 (1.247-4.109) | 0.007 |
| Previous MI | 2.134 (1.433-3.177) | <0.001 |  | 1.637 (1.043-2.568) | 0.032 |
| LVEF, % | 0.976 (0.965-0.987) | <0.001 |  | 0.986 (0.975-0.998) | 0.022 |
| eGFR, mL/min/1.73 m^2^ | 0.975 (0.969-0.981) | <0.001 |  | 0.985 (0.978-0.992) | <0.001 |
| STEMI | 1.177 (0.872-1.588) | 0.287 |  | 1.336 (0.954-1.869) | 0.091 |
| Cardiogenic shock | 2.009 (1.397-2.890) | <0.001 |  | 1.384 (0.908-2.110) | 0.131 |
| 3-vessel disease | 1.596 (1.184-2.152) | <0.001 |  | 1.364 (0.991-1.878) | 0.057 |

CI = confidence interval; eGFR = estimated glomerular filtration rate; HF = heart failure; HR = hazard ratio; LVEF = left ventricular ejection fraction; MACE = major adverse cardiovascular events; MI = myocardial infarction; SMuRF = standard modifiable cardiovascular risk factor; STEMI = ST-segment elevation myocardial infarction;

**Supplemental Table 7 Cox Proportional Hazards Analysis for MACE**

| Variable | Multivariable | |
| --- | --- | --- |
|  | HR (95% CI) | P value |
| Model 1 |  |  |
| Hypertension and diabetes | 1.264 (1.016-1.571) | 0.035 |
| Model 2 |  |  |
| Hypertension and current smoker | 1.190 (0.929-1.524) | 0.169 |
| Model 3 |  |  |
| Hypertension and dyslipidemia | 1.161 (0.925-1.458) | 0.199 |
| Model 4 |  |  |
| Hypertension, diabetes, dyslipidemia, and current smoker | 1.087 (0.932-1.268) | 0.286 |

CI = confidence interval; HR = hazard ratio; MACE = major adverse cardiovascular events.
